# Combined radiomics, PI-RADS, and clinical model improve significant prostate cancer prediction and guide biopsy decision

**DOI:** 10.1186/s13244-026-02295-4

**Published:** 2026-04-25

**Authors:** Andreu Antolin, Richard Mast, Nuria Roson, Javier Arce, Ramon Almodovar, Roger Cortada, Anna Alberti, Berta Miro, Olga Mendez, Almudena Maceda, Esther Serrano, Carmen Prieto-de-la-Lastra, Ana Jimenez-Pastor, Anna Nogué-Infante, Manuel Escobar, Enrique Trilla, Juan Morote

**Affiliations:** 1 Department of Radiology, Institut de Diagnòstic per la Imatge (IDI), Hospital Universitari Vall d’Hebron Passeig de la Vall d’Hebron, 119-129, 08035 Barcelona, Spain; 2 Department of Surgery, Universitat Autònoma de Barcelona (UAB) Plaça Cívica, 08193 Bellaterra, Barcelona Spain; 3 Department of Radiology, Hospital Universitari Vall d’Hebron Passeig de la Vall d’Hebron, 119-129, 08035 Barcelona, Spain; 4 Head of Radiology Department, Hospital Universitari Vall d’Hebron Passeig de la Vall d’Hebron, 119-129, 08035 Barcelona, Spain; 5 Statistics and Bioinformatics Department, Vall d’Hebron Research Institute (VHIR) Passeig de la Vall d’Hebron, 119-129, 08035 Barcelona, Spain; 6 Group of Biomedical Research in Urology, Vall Hebron Research Institute (VHIR) Passeig de la Vall d’Hebron, 119-129, 08035 Barcelona, Spain; 7 Vall d’Hebron Research Institute (VHIR) Passeig de la Vall d’Hebron, 119-129, 08035 Barcelona, Spain; 8 Quantitative Imaging Biomarkers in Medicine, Quibim S.L. Avenida Aragón 30 13th floor, office I–J, 46021 Valencia, Spain; 9 Clinical Director of Radiology Department, Hospital Universitari Vall d’Hebron Passeig de la Vall d’Hebron, 119-129, 08035 Barcelona, Spain; 10 Head of Department of Urology, Hospital Universitari Vall d’Hebron Passeig de la Vall d’Hebron, 119-129, 08035 Barcelona, Spain; 11 Department of Urology, Hospital Universitari Vall d’Hebron Passeig de la Vall d’Hebron, 119-129, 08035 Barcelona, Spain

**Keywords:** Prostatic neoplasms, Magnetic resonance imaging, Radiomics, Models (Statistical)

## Abstract

**Objectives:**

The aim of this study was to develop and validate an MRI radiomics-based predictive model to discriminate significant prostate cancer (sPCa), compare it with PI-RADS, and determine whether incorporating PI-RADS and other clinical variables improves clinical performance.

**Materials and methods:**

A retrospective observational study was conducted using a cohort of 1497 MRI cases from 1395 men to develop the models. For each case, the index-lesion PI-RADS score, systematic ± targeted biopsy results, and six additional clinical variables were collected. Prostate biopsy samples served as the reference standard, defining sPCa as Gleason Grade ≥ 7. Handcrafted radiomic features were extracted from automatically segmented prostate glands. Four machine learning models were developed: (1) Radiomics, (2) PI-RADS, (3) PI-RADS + Radiomics, and (4) PI-RADS + Radiomics + Clinical Variables. Model performance and comparisons were evaluated using the area under the curve (AUC), while clinical utility was assessed through the decision curve analysis plot, Clinical Utility plot, and the number of avoided biopsies.

**Results:**

The radiomics model did not perform significantly better than PI-RADS in the validation cohort (AUC 0.838 vs. 0.833, *p* = 0.874). The combination of radiomics, PI-RADS, and clinical variables achieved the highest performance, with an AUC of 0.891 (95% CI: 0.853–0.930), significantly outperforming the other models (*p* < 0.05). It also showed the highest specificity (29.41%) and biopsy avoidance rate (18.15%), although the differences were not statistically significant (*p* = 0.313).

**Conclusions:**

Incorporating radiomics and clinical variables into PI-RADS enhances its ability to discriminate sPCa, potentially decreasing false positives and unnecessary biopsies.

**Critical relevance statement:**

The incorporation of clinical variables and radiomics into PI-RADS enhances its ability to predict significant prostate cancer, helping mitigate some of PI-RADS’s current limitations, such as a significant false-positive rate, and might help reduce unnecessary biopsies.

**Key Points:**

- PI-RADS limitations result in overdiagnosis of indolent prostatic lesions and unnecessary biopsies.
- Radiomics and clinical variables enhance PI-RADS ability to detect significant prostate cancer.
- Combined clinical-radiological models reduce false positives and help avoid unnecessary biopsies.

**Graphical Abstract:**

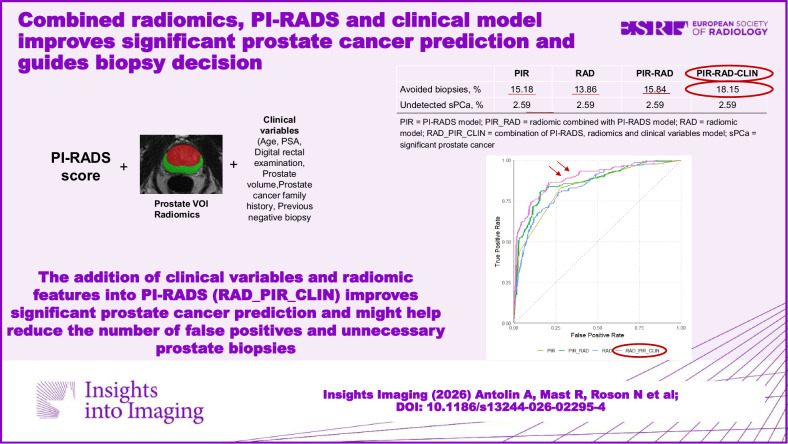

## Introduction

Prostate cancer (PCa) is the second most common malignancy and the fifth leading cause of cancer death in men [1]. Based on the International Society of Urological Pathology (ISUP) Gleason Grading [2], PCa is classified as indolent PCa (iPCa; ISUP 1/Gleason 6) or significant (sPCa; ISUP > 1/Gleason ≥ 7). iPCa is usually managed with active surveillance, while sPCa requires treatment due to a poorer prognosis.

Early detection of sPCa relies on multiparametric or biparametric prostate magnetic resonance imaging (mpMRI/bpMRI) in men with a serum prostate-specific antigen (PSA) > 3.0 ng/mL and/or abnormal digital rectal examination (DRE). Suspicious lesions are identified through the Prostate Imaging-Reporting and Data System (PI-RADS), currently in version 2.1, which provides a semiquantitative risk assessment of sPCa [3]. PI-RADS ≥ 3 lesions undergo targeted biopsies, complemented with a systematic prostate biopsy [4].

PI-RADS has limitations [5], including significant inter-reader variability [6, 7]. Its positive predictive value for PI-RADS 3 is 20%, leading to unnecessary biopsies and overdiagnosis of iPCa [8]. New predictive biomarkers are needed to reduce false positives [9].

Radiomics have shown promising results, but published studies have methodological limitations in data selection, model development, and validation [10–15]. Therefore, new radiomic models should be developed, compared with PI-RADS, and integrated with clinical variables to evaluate their added predictive value.

This study aims to: (1) develop and validate a radiomics-based predictive model for sPCa using MRI prostate segmentations in men with clinical suspicion of PCa; (2) compare it with PI-RADS, and develop/validate a combined model; (3) build and validate a multivariable model incorporating PI-RADS, radiomic features, and clinical variables, to assess improvements over previous models

## Materials and methods

### Study design

An observational retrospective study was conducted to develop, validate and compare different predictive models (see Model Development subsection) according to the study aims, following the Standards for the Reporting of Studies of Diagnostic Accuracy (STARD) [16].

Inclusion criteria were: (1) men with suspected PCa defined as serum PSA > 3 ng/mL and/or abnormal DRE; (2) prostate bpMRI or mpMRI reported following PI-RADS v2 [17] or v2.1 [3]; (3) prostate biopsy performed according to the standard protocol, consisting of targeted biopsy of suspicious lesions (PI-RADS ≥ 3) complemented by systematic biopsies, or systematic biopsies alone in PI-RADS < 3 but high clinical suspicion of sPCa; (4) availability of clinical variables (see Clinical Variables subsection).

Exclusion criteria were: (1) prior diagnosis of PCa and/or men in active surveillance; (2) prostate biopsies before MRI; (3) prostate biopsies performed later than 3 months after MRI; (4) missing information regarding MRI, clinical variables or prostate biopsies; (5) MRI artifacts that prevent an accurate reading.

1497 MRIs from 1395 men with suspected PCa were consecutively selected according to the inclusion/exclusion criteria from November 2015 to November 2022 at Vall Hebron Hospital, Barcelona, Spain. MRIs were reviewed by an abdominal radiologist with 5 years of experience, supervised by a senior radiologist with > 10 years of experience in prostate imaging.

The study was conducted in accordance with Checklist for Artificial Intelligence in Medical Imaging (CLAIM) guidelines [18].

### Ethical issues

The study was approved by the institutional Ethics Committee (PR(AG)02/2021). Data were collected and pseudonymized in accordance with ethical committee approval, using a unique code per case. The Ethics Committee granted a waiver of written informed consent, as participants were verbally informed at the time of specimen collection about the research use of their data. A Data Protection Impact Assessment was completed and reviewed by the committee.

### Image acquisition protocol and image pre-processing

MRI images were acquired according to PI-RADS and European Society of Urogenital Radiology guidelines [3, 17, 19]. Studies were performed in 1.5-T (Siemens MAGNETOM *Symphony*, Siemens Healthineers) and 3-T (Siemens MAGNETOM *Trio*, Siemens Healthineers) scanners. Biparametric studies consisted of T2, diffusion-weighted imaging (DWI) and apparent diffusion coefficient (ADC) sequences, while multiparametric studies included an additional dynamic contrast-enhanced (DCE) sequence. Between late 2018 and early 2019, mpMRI was progressively replaced by bpMRI for early diagnosis of PCa due to better cost-efficiency, absence of intravenous contrast, and comparable performance [20, 21]. Acquisition parameters are provided in Supplementary Tables S1 and S2. A phased array body coil was used in all.

Axial T2 and DWI sequences were selected, while DCE sequences were excluded since there is no clear evidence that quantitative features extracted from this sequence have added value in radiomic models [10]. Images were analyzed with QP-Prostate^®^ (Quibim S.L.), which is a medical device software (SaMD) with CE (*Conformité Européenne)*, UKCA (United Kingdom Conformity Assessed) and FDA 510k (Food and Drug Administration) certification. This software automatically segments peripheral and transitional (including the central zone) zones and seminal vesicles. It also provides the required image pre-processing steps prior to feature extraction to ensure reproducibility across patients (see Supplementary Material) and generates a new ADC map and DWI b-1400 sequence.

### Extraction of radiomic features

Handcrafted radiomic features were extracted using the QP-Insights® platform (Quibim S.L.) from the peripheral and transitional volume of interest (VOI) masks from T2 and generated DWI b1400/ADC maps. Seminal vesicle masks were discarded since prostate carcinoma is not generated in this region. 1379 radiomic features were obtained from each mask (see Supplementary Material). Therefore, 8274 characteristics were obtained per case, corresponding to 1379 features extracted from each VOI across the three sequences.

### Clinical variables

Seven clinical variables were collected according to the Barcelona Predictive Model of sPCa [22], which were obtained from the patient’s medical records. These were: age (years), serum PSA (ng/mL), prostate volume (mL, based on MRI), PI-RADS index lesion score (1–5), DRE (abnormal or normal), previous negative biopsy (yes/no) and previous family history of prostate cancer (yes/no). Clinical variables were preprocessed in accordance with the requirements of the algorithms used for model development. There were no missing values in the dataset.

### Reference standard

The presence or absence of sPCa in the prostatic biopsy samples was used as the outcome variable for this binary classification task. It was defined as ISUP > 1 (Gleason score ≥ 7), while non-sPCa included ISUP 1 (Gleason score 6) and benign lesions [2].

### Prostate biopsy protocol

Men with ≥ 1 suspicious MRI lesion (PI-RADS ≥ 3) underwent 2 to 4-core targeted biopsy per suspicious lesion, complemented with 12-core systematic biopsy. PI-RADS < 3 cases underwent only 12-core systematic biopsy. Targeted biopsies were performed using cognitive fusion with a transrectal ultrasound (TRUS) approach between 2015 and 2020, and software fusion with transperineal ultrasound (TPUS) from 2021 onward. All procedures were carried out by a single urologist with > 10 years of experience in prostate biopsy.

Tissue samples were examined by a dedicated genitourinary pathologist with > 15 years of experience.

### Model development

Four classification models were developed to assess the aim of the study: (1) PI-RADS model (PIR); (2) radiomics model (RAD); (3) radiomics + PI-RADS model (PIR_RAD); and (4) radiomics + PI-RADS + clinical variables model (RAD_PIR_CLIN). Model development is detailed in Supplementary Material.

The final models were also analyzed using SHAP (SHapley Additive exPlanations) to assess the contribution of individual features to the predictions [23], thereby enhancing model interpretability and explainability.

### Statistical analysis

Quantitative variables were summarized using mean and standard deviation (SD), as well as median and interquartile range (IQR). Categorical variables were reported as absolute and relative frequencies. Mean comparisons between partitions were performed using the independent samples of Student’s t-test when normality was met, or the Wilcoxon rank-sum test otherwise. Statistical differences in categorical variables between distributions were assessed using the chi-squared test. Discrimination of the four predictive models was evaluated based on the area under the Receiver Operating Characteristic (ROC) curve (AUC) with 95% confidence intervals (CIs). Additionally, the following metrics were calculated using a fixed probability threshold of 0.5: (1) accuracy; (2) balanced accuracy; (3) precision; and (4) F1-score. AUCs were compared using DeLong’s test. Decision curve analysis (DCA) was performed to evaluate net benefit, and clinical effectiveness was assessed through the clinical utility curve (CUC), representing the rates of avoided prostate biopsies and the undetected rate of sPCa according to the continuous probability threshold.

To ensure comparability, all models were evaluated at a fixed sensitivity of 97.4%, corresponding to the PI-RADS ≥ 3 threshold. For each model, the predicted probability threshold was adjusted accordingly, and biopsy avoidance was defined as the proportion of patients without sPCa whose predicted probability fell below this threshold. To statistically compare model performance against the PI-RADS baseline in terms of specificity and biopsy avoidance, McNemar’s test was applied. The analysis was restricted to patients without sPCa to assess whether the specificity improvements observed with the alternative models were statistically significant.

All statistical tests were two-sided, and a *p*-value < 0.05 was considered indicative of statistical significance.

Statistical analysis was done with R software version 4.5.0 (R Foundation for Statistical Computing).

## Results

### Clinical characteristics of the cohorts

1497 MRI cases from 1395 men with suspected PCa were consecutively selected according to the inclusion/exclusion criteria. The cohort was randomly split into a training cohort (80%) and a test cohort (20%). The clinical characteristics of the overall cohort and each partition are summarized in Table 1. The prevalence of sPCa was 39.2%. PSA had an SD of 130.4 ng/mL since the minimum and maximum values were 0.24 and 4414.4 ng/mL, respectively. 77.7% were biopsy naive, while 22.3% had a previous negative prostate biopsy. No statistically significant differences were observed between the training and test cohorts in any of the assessed variables, including sPCa detection rate and PI-RADS score of the index lesion.

**Table 1:** Clinical characteristics of the entire cohort and the respective training and test partitions

Clinical characteristics	Entire cohort	Training cohort	Test cohort	*p*-value
Number of cases	1497	1194	303	-
Mean age, years (SD) Median age, years [IQR]	68.5 (8.4) 69.1 (62.8, 74.1)	68.5 (8.3) 69 (62.8, 74.2)	68.5 (8.7) 69.1 (63, 74)	0.719
Mean serum PSA, ng/mL (SD) Median serum PSA, ng/mL (IQR)	13.7 (116.6) 6.1 (4.3, 9.8)	14.7 (130.4) 6.1 (4.3, 9.9)	9.6 (14.9) 5.8 (4.3, 9.5)	0.382
Abnormal DRE, *n* (%)	313 (20.8)	256 (21.4)	56 (18.5)	0.292
PCa family history, *n* (%)	103 (6.9)	86 (7.2)	17 (5.6)	0.395
Previous negative prostate biopsy, *n* (%)	334 (22.3)	268 (22.4)	66 (21.8)	0.865
Mean prostate volume, mL (SD) Median prostate volume, mL (IQR)	61.7 (31.5) 55 (40, 76)	61.7 (31.2) 55 (40, 76)	61.5 (32.6) 54 (40, 76)	0.617
PI-RADS score of index lesion, *n* (%)				0.293
1	131 (8.8)	100 (8.4)	31 (10.2)	
2	61 (4.1)	43 (3.6)	18 (5.9)	
3	526 (35.1)	420 (35.2)	106 (35)	
4	477 (31.9)	386 (32.3)	91 (30)	
5	302 (20.2)	245 (20.5)	57 (18.8)	
sPCa detection rate, *n* (%)	577 (39.2)	471 (39.4)	116 (38.3)	0.761

Data of quantitative variables is represented in mean and standard deviation (SD) at the upper part of the row, as well as median and interquartile range (IQR) at the bottom. Data of categorical variables is represented in absolute frequency (*n*) and relative frequency in %

*DRE* digital rectal examination, *IQR* interquartile range, *n* absolute frequency, *PCa* prostate cancer, *PI-RADS* Prostate Imaging-Reporting and Data System, *PSA* prostate-specific antigen, *SD* standard deviation, *sPCa* significant prostate cancer

The CLAIM checklist is depicted in Supplementary Material Table S3.

### Model performance in discriminating sPCa

**Fig. 1:**
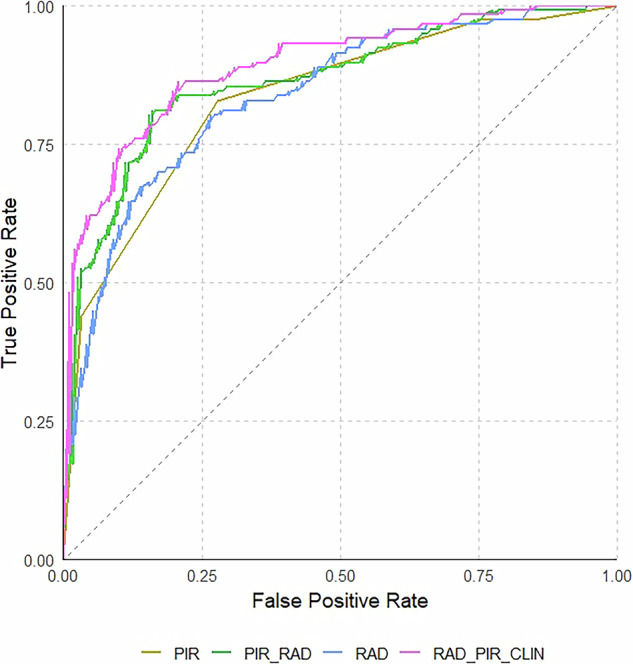
Receiver operating characteristics (ROC) curves showing the discrimination ability for significant prostate cancer (sPCa) of the PI-RADS model (PIR), radiomics model (RAD), PI-RADS +  radiomics model (PIR_RAD), and radiomics + PI-RADS + clinical variables model (RAD_PIR_CLIN)

Random Forest was the algorithm that yielded the best overall performance among all classifiers tested. The AUC values for each Random Forest-based model, as well as the PI-RADS logistic regression model, are summarized in Table 2, which also includes *p*-values from pairwise comparisons between each model and the subsequent one. The ROC curve of each model is plotted in Fig. 1.

**Table 2:** Models’ performance with pairwise comparisons between each model and the subsequent one

Classification model	AUC (95%, CI)	*p*-value	Holm adjusted *p*-value
PI-RADS (PIR)	0.833 (0.788–0.878)	-	-
Radiomics (RAD)	0.838 (0.792–0.884)	0.874	0.874
PI-RADS-Radiomics (PIR_RAD)	0.863 (0.818–0.907)	0.236	0.472
Radiomics–PI-RADS–Clinical Variables (RAD_PIR_CLIN)	0.891 (0.853–0.930)	0.003	0.012

*AUC* area under the curve, *CI* confidence interval

The PIR model achieved an AUC of 0.833 (95% CI: 0.788–0.878), while the RAD model reached an AUC of 0.838 (95% CI: 0.792–0.884), with no statistically significant difference between them in sPCa detection (*p* = 0.874). The combination of PI-RADS and radiomic features (PIR_RAD) showed a higher AUC of 0.863 (95% CI: 0.818–0.907) but was not significantly superior over both individual models (*p* = 0.05 and *p* = 0.874 for PIR and RAD, respectively). Finally, the addition of clinical variables to the combined PI-RADS and radiomics model (RAD_PIR_CLIN) resulted in an AUC of 0.891 (95% CI: 0.853–0.930), which was significantly higher than the PIR, RAD and PIR_RAD models (*p* < 0.05 for each comparison).

The accuracy, balanced accuracy, precision, and F1-score for RAD, PIR_RAD and RAD_PIR_CLIN, considering a threshold of 0.5, are presented in Table S7.

### Net benefit

**Fig. 2:**
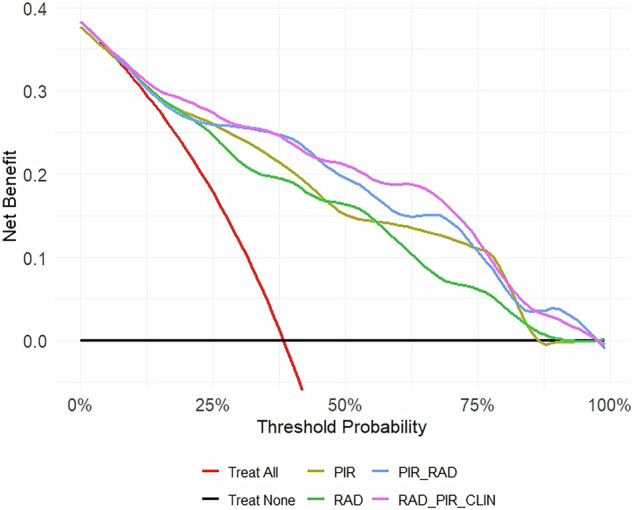
Decision curve analysis (DCA) showing the net benefit of the PI-RADS model (PIR), radiomic model (RAD), PI-RADS + radiomic model (PIR_RAD), and the radiomic + PI-RADS + clinical variables model (RAD_PIR_CLIN) compared to performing biopsy on all or none men with prostate cancer suspicion

The DCA of the four models is plotted in Fig. 2. The PIR_RAD model and the addition of clinical variables (PIR_RAD_CLIN) showed the highest net clinical benefit in the widest range of threshold probabilities (approximately 30–60%). The PIR and RAD model showed a lower net clinical benefit.

### Clinical efficacy

The sensitivity of all models was fixed to match that of the PI-RADS ≥ 3 reference threshold, which in this dataset was 97.40%. This ensures a fair comparison in terms of specificity and biopsy avoidance, consistent with current clinical practice that recommends targeted biopsy for lesions scored PI-RADS ≥ 3. Table 3 summarizes the resulting diagnostic metrics for each model based on this supposition. The PIR model achieved a specificity of 24.60% and a biopsy avoidance rate of 15.18%. The RAD model showed slightly lower specificity (22.46%) and biopsy avoidance (13.86%), with no statistically significant difference compared to PIR (*p* = 0.711). The combined PIR_RAD model improved the specificity (25.67%) and biopsy avoidance (15.84%) over both standalone models but did not achieve statistical significance in comparison to PIR (*p* = 0.896). The addition of clinical variables further improved specificity and biopsy avoidance in comparison to the previous models (29.41% and 18.15%), but the difference was not statistically significant (*p* = 0.313). Importantly, the proportion of undetected sPCa remained constant across all models (2.59%) due to the fixed sensitivity.

**Table 3:** Comparison of model clinical efficacy at fixed sensitivity, following recommendations to avoid biopsies for PI-RADS < 3

	PIR	RAD	PIR-RAD	PIR-RAD-CLIN
Threshold, %	10.90	13.82	8.50	10.45
True positive, *n*	113	113	113	113
False negatives, *n*	3	3	3	3
True negative, *n*	46	42	48	55
False positives, *n*	141	145	139	132
Sensitivity, %	97.41	97.41	97.41	97.41
Specificity, %	24.60	22.46	25.67	29.41
Youden Index, %	22.01	19.87	23.08	26.83
PPV, %	44.49	43.80	44.84	46.12
NPV, %	93.89	93.33	94.11	94.83
Avoided biopsies, %	15.18	13.86	15.84	18.15
Undetected sPCa, %	2.59	2.59	2.59	2.59
*p*-value	-	0.711	0.896	0.314

*NPV* negative predictive value, *PIR* PI-RADS model, *PIR_RAD* radiomic combined with PI-RADS model, *PPV* positive predictive value, *RAD* radiomic model, *RAD_PIR_CLIN* combination of PI-RADS, radiomics and clinical variables model, *sPCa* significant prostate cancer

**Fig. 3:**
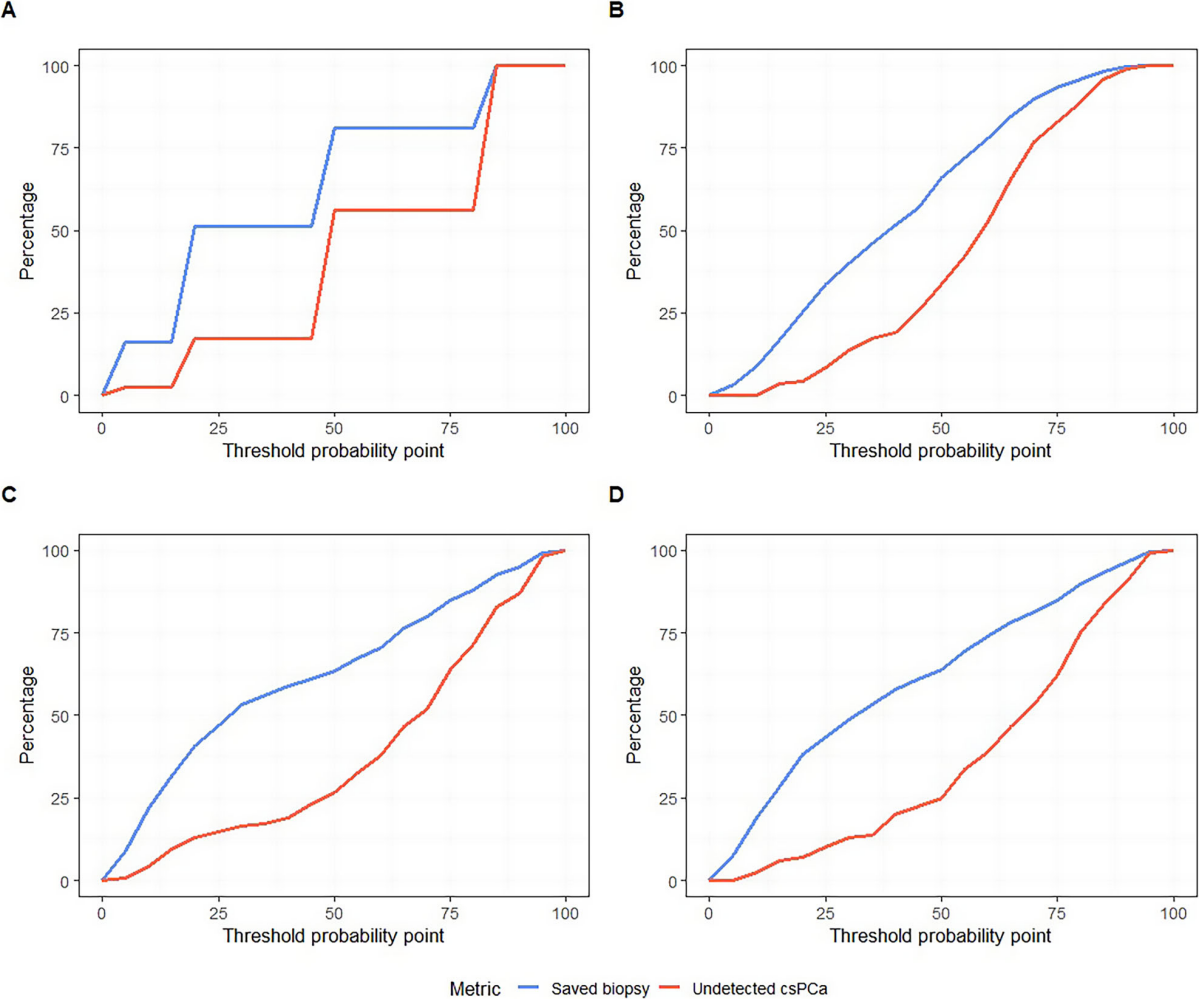
Clinical utility curve (CUC) showing the proportion of biopsies that could be avoided and the proportion of undetected sPCa across a range of threshold probabilities for performing targeted biopsy, in the PI-RADS model (**A**), radiomic model (**B**), PI-RADS + radiomic model (**C**), and PI-RADS +  radiomic + clinical variables (**D**)

The CUC for the four models is presented in Fig. 3, showing the balance between the proportion of biopsies that could be avoided and the proportion of undetected sPCa across a range of threshold probabilities for performing targeted biopsy. Like the DCA results, the PIR_RAD model and the addition of clinical variables (RAD_PIR_CLIN) allowed for a greater number of biopsies to be safely avoided while maintaining similar or lower rates of undetected sPCa compared to the other two models.

### Explainability

**Fig. 4:**
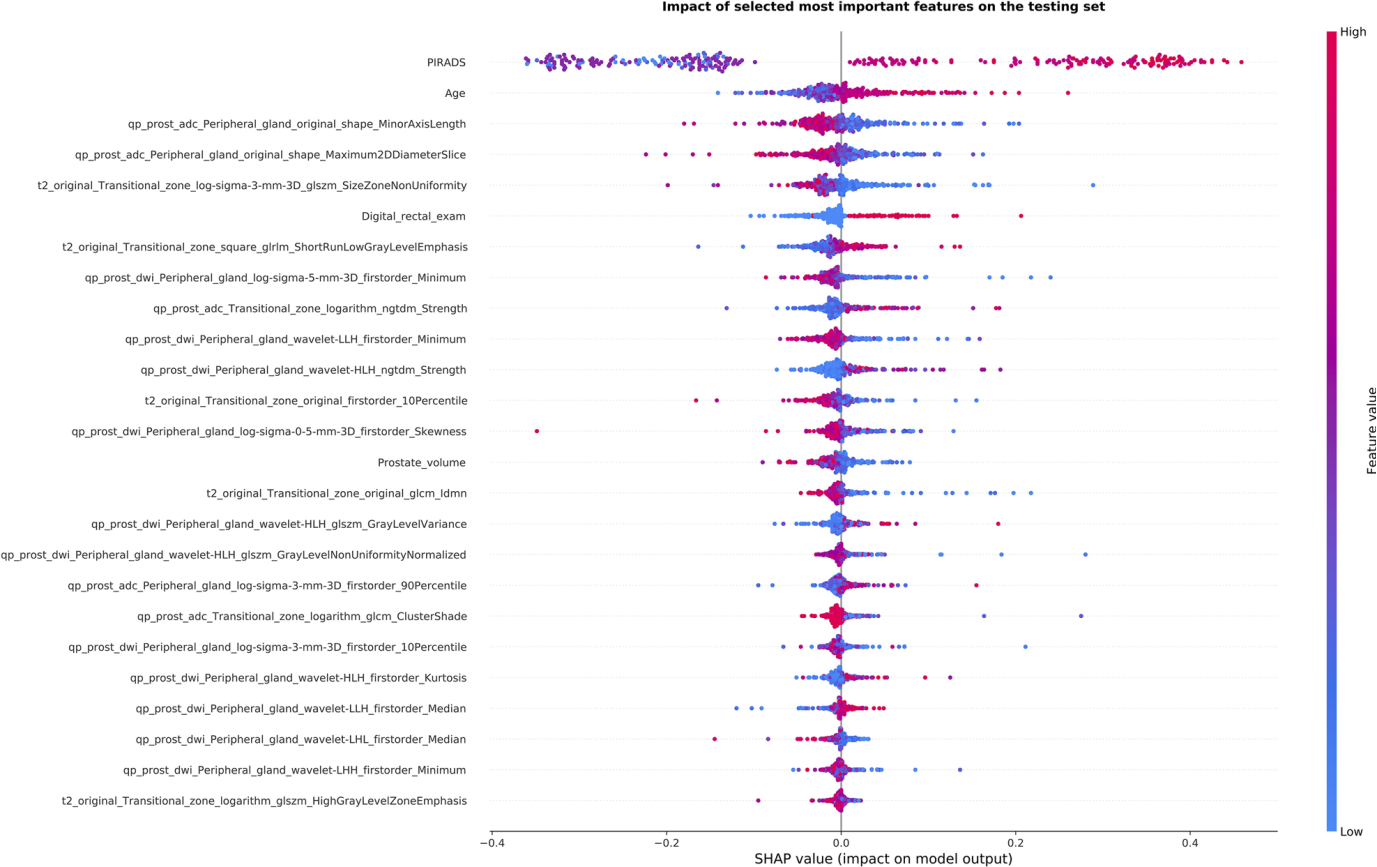
SHapley Additive exPlanations (SHAP) summary plot showing the top 25 features contributing to significant prostate cancer (sPCa) prediction in the PI-RADS + radiomics + clinical variables model (RAD_PIR_CLIN)

SHAP analysis was applied to the RAD_PIR_CLIN model, which combined 25 variables to predict the presence or absence of sPCa (Fig. 4).

PI-RADS score was the most influential predictor in the model, followed by patient age. Higher values of these two variables were associated with an increased risk of sPCa. Prostate volume showed an inverse correlation, with larger volumes associated with a lower chance of sPCa. Radiomic features showed variable effects depending on their values. Those derived from DWI b-1400 and ADC sequences showed greater and more consistent positive impact on model predictions of sPCa.

False positives were associated with higher PI-RADS scores, suggesting that imaging-based suspicion is important in model prediction, in agreement with the SHAP analysis. This dependence suggests that model performance may be influenced by reader expertise, potentially performing better in experienced centers.

False negatives were more frequent in patients with larger prostate volumes, which may reflect reduced lesion conspicuity in larger glands. This could affect PI-RADS assessment and potentially influence radiomic feature extraction.

## Discussion

A handcrafted radiomic model based on prostate segmentations was developed to discriminate sPCa in bpMRI from men with suspected PCa. The radiomic and PI-RADS models achieved moderate and comparable performance in discriminating sPCa. Combining PI-RADS and radiomic features yielded slightly higher performance but was not significantly superior to either model alone. Adding clinical variables further improved discrimination and achieved statistical significance when compared to the other models.

Our results are consistent with the published literature, which shows considerable variability, as noted in previous systematic reviews and meta-analyses [10–12, 15]. Methodological limitations, including small cohorts, non-uniform reference standards, manual segmentation variability, and the lack of multimodal models, contribute to this variability and hinder the true potential of radiomics. In this study, we mitigated some of these limitations by using a large cohort, a clear biopsy-based reference standard, automatic prostate segmentation, and multimodal models integrating clinical features to improve sPCa discrimination.

Clinical implementation of radiomic models is partially halted by the lack of evidence comparing these models with PI-RADS [15]. In our study, no significant differences were observed between the radiomic and PI-RADS models, consistent with previous reviews [15].

However, differences might emerge when radiologist experience is considered. Bao et al [24] reported better performance in junior radiologists who used artificial intelligence (AI), but not clearly in senior/expert readers. Similarly, Hamm et al [25] found improved performance among nonexperts, particularly in PI-RADS 3 lesions. Other studies reported no differences when compared to expert radiologists [26]. In contrast, a recent multicenter study including over 9000 patients and more than 60 radiologists (with a median of 7 years of experience) reported significantly better performance of the AI model compared to radiologists, although the model incorporated clinical metadata [27]. However, in a multidisciplinary setting where radiologists had access to full medical records, the AI model was neither significantly superior nor inferior to radiologists [27]. Prospective studies are needed to clarify the potential of radiomic models.

The combination of radiomic features and PI-RADS yielded better but non-significant discriminating capacity than the models alone, consistent with available literature [15, 28]. The addition of clinical variables significantly improved performance in comparison to the rest of the models. This might support the integration of these models as a complementary tool to aid the radiologist, which might help overcome some of the limitations of PI-RADS v2.1 [5] and inherent inter-reader variability in MRI interpretation [6, 7].

Age, DRE, PSA, prostate volume, and biopsy status (biopsy-naïve vs. previously biopsied) are among the clinical variables commonly incorporated into sPCa risk models [22, 29–31]. Prior studies have shown that adding these variables to PI-RADS enhances the detection of sPCa and helps reduce unnecessary biopsies [22]. The combined PI-RADS and radiomics model, as well as the inclusion of clinical variables, demonstrated a higher net clinical benefit across a wide range of threshold probabilities compared to standalone models, in line with previous findings [24, 32]. This improvement correlates with a slightly higher specificity and biopsy avoidance observed in the combined models, when evaluated at a fixed sensitivity. However, the differences were not statistically significant. A recent study reported that the integration of deep radiomics, PI-RADS, and clinical variables improved patient risk stratification prior to biopsy and led to a reduction in unnecessary procedures [32]. Further studies with larger cohorts or alternative methodological approaches are needed to fully assess this potential, as overdiagnosis of non-sPCa lesions remains a challenge [9].

There were several limitations in this study. First, a retrospective single-center and single-vendor cohort was used, which might limit model generalizability, although some studies reported no significant differences based on magnetic field strength or MRI vendor [33, 34]. Second, PI-RADS scoring was based on both versions 2.0 and 2.1, which may introduce bias. However, it remains unclear whether these differences have a significant clinical impact [35, 36]. Third, deep radiomics and lesion-mask feature extraction were not tested. Studies using whole-prostate segmentations on large datasets have shown comparable results to those based on lesion-specific segmentations [27, 32]. These studies were based on deep radiomics, so the use of handcrafted radiomics might limit radiomics’ performance. This is consistent with past reviews that favored deep learning techniques [12]. We believe deep radiomics models will continually outperform handcrafted ones with increasingly larger datasets. Lastly, no external validation was performed due to the difficulty in obtaining the clinical variables in external cohorts.

In conclusion, the addition of radiomics and clinical variables to PI-RADS improves its discriminatory capacity for detecting sPCa, potentially reducing false positives and unnecessary biopsies in men with suspected PCa. However, further methodological refinement and prospective validation studies are necessary to fully establish the clinical impact and support the integration of such models into routine clinical practice.

## Supplementary information

ELECTRONIC SUPPLEMENTARY MATERIAL

## Data Availability

The datasets used and/or analyzed during the current study are available from the corresponding author on reasonable request.

## Abbreviations

ADC: Apparent diffusion coefficient
AI: Artificial intelligence
AUC: Area under the curve
bpMRI: Biparametric prostate MRI
CI: Confidence intervals
CUC: Clinical utility curve
DCA: Decision curve analysis
DCE: Dynamic contrast-enhanced
DRE: Digital rectal examination
DWI: Diffusion-weighted imaging
iPCa: Indolent prostate cancer
IQR: Interquartile range
ISUP: International Society of Urological Pathology
mpMRI: Multiparametric prostate MRI
MRI: Magnetic resonance imaging
PCa: Prostate cancer
PIR: PI-RADS model
PIR_RAD: Radiomics + PI-RADS model
PI-RADS: Prostate Imaging-Reporting and Data System
PSA: Prostate-specific antigen
RAD: Radiomics model
RAD_PIR_CLIN: Radiomics + PI-RADS + Clinical Variables model
SD: Standard deviation
SHAP: SHapley Additive exPlanations
sPCa: Significant prostate cancer
VOI: Volume of interest
