# Correction: Combined radiomics, PI-RADS, and clinical model improve significant prostate cancer prediction and guide biopsy decision

**DOI:** 10.1186/s13244-026-02313-5

**Published:** 2026-06-02

**Authors:** Andreu Antolin, Richard Mast, Nuria Roson, Javier Arce, Ramon Almodovar, Roger Cortada, Anna Alberti, Berta Miro, Olga Mendez, Almudena Maceda, Esther Serrano, Carmen Prieto-de-la-Lastra, Ana Jimenez-Pastor, Anna Nogué-Infante, Manuel Escobar, Enrique Trilla, Juan Morote

**Affiliations:** 1 Department of Radiology, Institut de Diagnòstic per la Imatge (IDI), Hospital Universitari Vall d’Hebron Passeig de la Vall d’Hebron, 119-129, Barcelona, Spain; 2 Department of Surgery, Universitat Autònoma de Barcelona (UAB) Plaça Cívica, Barcelona, Spain; 3 Department of Radiology, Hospital Universitari Vall d’Hebron Passeig de la Vall d’Hebron, 119-129, Barcelona, Spain; 4 Head of Radiology Department, Hospital Universitari Vall d’Hebron Passeig de la Vall d’Hebron, 119-129, Barcelona, Spain; 5 Statistics and Bioinformatics Department, Vall d’Hebron Research Institute (VHIR) Passeig de la Vall d’Hebron, 119-129, Barcelona, Spain; 6 Group of Biomedical Research in Urology, Vall Hebron Research Institute (VHIR) Passeig de la Vall d’Hebron, 119-129, Barcelona, Spain; 7 Vall d’Hebron Research Institute (VHIR) Passeig de la Vall d’Hebron, 119-129, Barcelona, Spain; 8 Quantitative Imaging Biomarkers in Medicine, Quibim S.L. Avenida Aragón 30 13th floor, office I–J, Valencia, Spain; 9 Clinical Director of Radiology Department, Hospital Universitari Vall d’Hebron Passeig de la Vall d’Hebron, 119-129, Barcelona, Spain; 10 Head of Department of Urology, Hospital Universitari Vall d’Hebron Passeig de la Vall d’Hebron, 119-129, Barcelona, Spain; 11 Department of Urology, Hospital Universitari Vall d’Hebron Passeig de la Vall d’Hebron, 119-129, Barcelona, Spain

**Correction to: Insights into Imaging**

10.1186/s13244-026-02295-4, published online 25 April 2026

In this article, the author’s name Carmen Prieto-de-la-Lastra was incorrectly written as Carmen Pietro-de-la-Lastra.

The original article has been corrected.

