# Supplementary material for: Combined radiomics, PI-RADS, and clinical model improve significant prostate cancer prediction and guide biopsy decision
Source: Insights Imaging. 2026 Apr 25;17:118. doi: 10.1186/s13244-026-02295-4 (PMC13110268; doi:10.1186/s13244-026-02295-4)
Supplement: Supplementary file 1 — ELECTRONIC SUPPLEMENTARY MATERIAL [file 13244_2026_2295_MOESM1_ESM.pdf]

Combined Radiomics, PI-RADS and Clinical Model Improves Significant Prostate Cancer Prediction and Guides Biopsy Decision

ELECTRONIC SUPPLEMENTARY MATERIAL

Image and Data Pre-processing. The preprocessing of T2 images included noise reduction using a non-local means filter, along with bias field correction to address intensity inhomogeneities. For the diffusion-weighted imaging (DWI) sequence, denoising was performed using the Perona-Malik anisotropic diffusion filter. Subsequently, all images were resampled to isotropic voxel spacing by selecting the smallest original voxel dimension across the x, y, and z axes as the target resolution. Segmentation masks were transformed into the new isotropic voxel space using nearest-neighbor interpolation. Finally, z-score normalization was applied to standardize image intensities.

The apparent diffusion coefficient (ADC) map was generated based on the DWI sequence using a Gaussian mono-exponential model by the software to calculate an extra DWI b-1400 sequence based on ADC and available DWI b-values.

Tabular data did not have missing values, so no missing data handling was required. Tabular data was also pre-processed according to the algorithm’s requirements (see subsection Model Development).

Radiomic Features. A total of 1379 radiomic features were obtained from each volume of interest (VOI) in each sequence:

- Shape Features (n=14): describe the geometric properties of the VOI, including surface area, volume, maximum diameter, elongation, sphericity, or surface-to-volume ratio.
- First-order Features (n=18): describe the distribution of voxel intensities within the VOI using histogram-based metrics such as energy, entropy, mean, interquartile range, skewness, kurtosis or uniformity.
- Second-order Features (n=73): also known as texture features. They capture statistical relationships between neighboring voxels, derived from several gray-level matrices: GLCM (Gray-Level Co-occurrence Matrix), GLRLM (Gray-Level Run Length Matrix), GLSZM (Gray-Level Size Zone Matrix), NGTDM (Neighboring Gray Tone Difference Matrix) and GLDM (Gray-Level Dependence Matrix).
- Higher-order Features (n=1274): extracted after applying image filters that highlight different aspects of intensity and texture such as square filter, exponential filter, logarithmic filter, wavelet filter (Haar) and Laplacian of Gaussian (LoG) filter.

Model Development. The dataset was randomly divided into 80% for training and 20% for testing. Five different machine learning algorithms were trained to identify the most effective methods for radiomic (RAD), radiomics + PI-RADS (PIR_RAD) and radiomics + PI-RADS + clinical variables model (RAD_PIR_CLIN) models for classifying significant prostate cancer (sPCa): a) K-Nearest Neighbors (KNN); b) Support Vector Classifier (SVC); c) Multilayer Perceptron (MLP); d) Decision Tree (DT); and e) Random Forest (RF). The PI-RADS-based classifier (PIR) was trained with a logistic regression (GLM) since it involved a single predictor.

A stratified split was performed to preserve the sPCa class balance across groups. All classifiers besides GLM were trained using a 5-fold cross-validation strategy combined with a grid-search-based hyperparameter tuning procedure.

The grid search for hyperparameters followed an iterative, coarse-to-fine approach. Initially, broad and general hyperparameter ranges were explored to determine the most suitable configurations for the classification task. Afterwards, finer hyperparameters were evaluated to optimize performance. Hyperparameter tuning was conducted strictly within the training folds, and the optimal configuration for each model was defined as the one achieving the highest cross-validated performance.

For each fold and classifier, a customizable preprocessing pipeline was applied, consisting of the following steps:

- Variable harmonization using ComBat: applied to radiomic features. Classifiers were trained with and without feature harmonization to evaluate the best configuration.
- Feature reduction via Pearson correlation filtering: applied to radiomic features.
- Feature selection using the Minimum Redundancy Maximum Relevance (mRMR) method.
- Z-score normalization of quantitative variables.
- Dimensionality reduction via Principal Component Analysis (PCA): applied to quantitative variables.
- One-hot encoding (OHE): applied to categorical variables depending on the classifier.
- Classifier training.

The final models were also analyzed using SHAP (SHapley Additive exPlanations) to assess the contribution of individual features to the predictions, thereby enhancing model interpretability and explainability.

In this analysis, each point represents a single prediction's SHAP value of a single prediction, with vertical jittering applied to enhance visibility. The color gradient indicates the feature's value, ranging from low (blue) to high (red). This plot highlights both the direction (positive or negative impact) and the distribution of SHAP values, revealing patterns and interactions that contribute to the model's predictions.

The following software/libraries were used for model development: Python v3.6, Python v3.7, numpy v1.19.5, pandas v0.25.3, seaborn v0.11.2, scikit-learn v0.24.2, neuroHarmonize v2.4.5, statsmodels v0.12.2, neuroCombat v.0.2.12, mrmr-selection v0.2.8 and shap v0.41.0.

Extraction of radiomic features was done with QP-Insights® platform (Quibim S.L., Valencia, Spain).

Model Calibration. Model calibration was assessed on the independent test set using calibration curves, comparing predicted probabilities with observed outcomes. For each model, predicted probabilities were grouped into equally sized bins, and the mean predicted probability was plotted against the observed event frequency. In addition, the Brier score and the expected calibration error (ECE) were reported. This analysis was performed for all final evaluated models.

Calibration curves for the evaluated models are shown in Figure S1. All models demonstrated a good agreement between predicted probabilities and observed outcomes on the test set. The PI-RADS-only logistic regression model showed a calibration curve close to the diagonal, indicating adequate probabilistic calibration despite the discrete nature of the input variable. Models incorporating radiomics features provided a smoother and more continuous risk distribution across the probability range.

Quantitative calibration metrics are also reported in Figure S1. The RAD_PIR_CLIN model achieved the lowest Brier score (0.129), indicating the best overall probabilistic accuracy.

Expected calibration error (ECE) values were low for all models, with the lowest ECE observed for the PI-RADS-only model (0.026).

This analysis provided relevant insights into the clinical behavior of the evaluated models. Models incorporating radiomics features showed consistent probabilistic calibration across the full range of predicted risks, supporting their suitability for individualized risk estimation.

The PI-RADS-only model exhibited adequate calibration when PI-RADS was used as a discrete predictor in a logistic regression framework. However, the limited number of input levels (PI-RADS 2–5) constrains the resolution of predicted probabilities. In contrast, radiomics-based models yielded more granular probability estimates while maintaining good calibration, which may be advantageous for individualized risk assessment and clinical decision-making.

Overall, these findings suggest that the integration of radiomics (and clinical variables) improves not only discrimination but also the reliability of predicted probabilities, which strengthens the practical relevance of the proposed approach.

Table S1. MRI parameters for T2, diffusion-weighted imaging (DWI) and Dynamic Contrast-Enhanced (DCE) according to the institutional protocol for 1.5T.

	**T2**	**Diffusion-weighted imaging (DWI)**	**Dynamic Contrast-Enhanced (DCE)**
Sequence type	Turbo-spin-echo (TSE)	RESOLVE (Readout Segmentation Of Long Variable Echo-trains)	T1-weighted VIBE (Volumetric Interpolated Breath-hold Examination)
Acquisition plane	Axial	Axial	Axial
Slice thickness (mm)	3	3	3
Number of slices	33	33	26
Field-of-view	200 x 200	220 x 220	240 x 240
Voxel size (mm^3)	0.3 x 0.3 x 3.0	0.9 x 0.9 x 3.0	1.3 x 1.3 x 3.0
Echo time (ms)	111	60	2.35
Repetition time (ms)	4930	6800	4.2
b-values (s/mm^2)	N/A	50/400/800/1400 (sometimes computed b1400)	N/A
Acquisition time (min)	5:35	7:10	3:20

Note: An optional protocol was also used in which DWI was also acquired using Siemens' ZOOMit technology, which applies 2D spatially selective radiofrequency excitation to achieve a reduced field of view and minimize geometric distortion. Acquisition plane: axial; Slice thickness (mm): 3mm; Number of slices: 33; Field-of-view: 200 x 200; Voxel size (mm^3): 0.8 x 0.8 x 3.0; Echo time (ms): 72; Repetition time (ms): 5700; b-values (s/mm^2): 50, 1000 and computed 2000; Acquisition time (min): 5:21.

Table S2. MRI parameters for T2, diffusion-weighted imaging (DWI) and Dynamic Contrast-Enhanced (DCE) according to the institutional protocol for 3T.

	**T2**	**Diffusion-weighted imaging (DWI)**	**Dynamic Contrast-Enhanced (DCE)**
Sequence type	Turbo-spin-echo (TSE)	RESOLVE (Readout Segmentation Of Long Variable Echo-trains)	T1-weighted VIBE (Volumetric Interpolated Breath-hold Examination)
Acquisition plane	Axial	Axial	Axial
Slice thickness (mm)	3	3	3
Number of slices	32	32	26
Field-of-view	180 x 180	240 x 240	240 x 240
Voxel size (mm^3)	0.2 x 0.2 x 3.0	2.2 x 2.2 x 3.0	1.3 x 1.3 x 3.0
Echo time (ms)	119	51	2.35
Repetition time (ms)	4360	3080	4.2
b-values (s/mm^2)	N/A		N/A
Acquisition time (min)	4:06	3:43	3:20

Note: An optional protocol was also used in which DWI was also acquired using Siemens' ZOOMit technology, which applies 2D spatially selective radiofrequency excitation to achieve a reduced field of view and minimize geometric distortion. Acquisition plane: axial; Slice thickness (mm): 3mm; Number of slices: 32; Field-of-view: 200 x 200; Voxel size (mm^3): 0.8 x 0.8 x 3.0; Echo time (ms): 79; Repetition time (ms): 4700; b-values (s/mm^2): 50, 1400 and computed 2000; Acquisition time (min): 5:31.

Table S3. Checklist for Artificial Intelligence in Medical Imaging (CLAIM). Source: Tejani AS, Klontzas ME, Gatti AA, et al (2024) Checklist for Artificial Intelligence in Medical Imaging (CLAIM):2024 Update. Radiol Artif Intell 6(4):e240300. https://doi.org/10.1148/ryai.240300

Checklist for Artificial Intelligence in Medical Imaging (CLAIM): 2024 Update

Section / Topic	No.	Item	Page / Line	No	NA
TITLE / ABSTRACT					
	**1**	Identification as a study of AI methodology, specifying the category of technology used (e.g., deep learning)	**Title**		
ABSTRACT					
	**2**	Summary of study design, methods, results, and conclusions	**Abstract**		
INTRODUCTION					
	**3**	Scientific and/or clinical background, including the intended use and role of the AI approach	**Paragraph 1 to 4**		
	**4**	Study aims, objectives, and hypotheses	**Paragraph 5**		
METHODS					
Study Design	**5**	Prospective or retrospective study	**Paragraph 1**		
	**6**	Study goal	**Paragraph 1**		
Data	**7**	Data sources	**Paragraph 4**		
	**8**	Inclusion and exclusion criteria	**Paragraph 2 to 3**		
	**9**	Data pre-processing	**Paragraph 7, 8, 10**		
	**10**	Selection of data subsets	**Suppl Material (subsection Model Development)**		
	**11**	De-identification methods	**Paragraph 6**		
	**12**	How missing data were handled	**Paragraph 10 and Suppl Material**		
	**13**	Image acquisition protocol	**Paragraph 7 and tables S1-S2**		

Reference Standard	**14**	Definition of method(s) used to obtain reference standard	**Paragraph 11 to 12**		
	**15**	Rationale for choosing the reference standard	**Paragraph 11**		
	**16**	Source of reference standard annotations	**Paragraph 11 to 12**		
	**17**	Annotation of test set	**Same as training set**		
	**18**	Measures of inter- and intra-rater variability of features described by the annotators			**NA**
Data Partitions	**19**	How data were assigned to partitions	**Suppl Material (subsection Model Development)**		
	**20**	Level at which partitions are disjoint	**Suppl Material (subsection Model Development)**		
Testing Data	**21**	Intended sample size		**No**	
Model	**22**	Detailed description of model	**Paragraph 14 (and subsection Model Development in Suppl Material)**		
	**23**	Software libraries, frameworks, and packages	**Paragraph 8, 9 19 and Suppl Material (subsection Model Development)**		
	**24**	Initialization of model parameters	**Suppl Material (subsection Model Development)**		
Training	**25**	Details of training approach	**Suppl Material (subsection**		

			**Model Development)**		
	**26**	Method of selecting the final model	**Suppl Material (subsection Model Development)**		
	**27**	Ensembling techniques			**NA**
Evaluation	**28**	Metrics of model performance	**Paragraph 16 to 18**		
	**29**	Statistical measures of significance and uncertainty	**Paragraph 16 to 18**		
	**30**	Robustness or sensitivity analysis		**No**	
	**31**	Methods for explainability or interpretability	**Paragraph 15 and Suppl Material (subsection Model Development)**		
	**32**	Evaluation on internal data	**Suppl Material (subsection Model Development)**		
	**33**	Testing on external data	**Discussion paragraph 7**		
	**34**	Clinical trial registration			**NA**
RESULTS					
Data	**35**	Numbers of patients or examinations included and excluded	**Only the final number**	**No**	
	**36**	Demographic and clinical characteristics of cases in each partition	**Paragraph 1 and Table 1**		
Model performance	**37**	Performance metrics and measures of statistical uncertainty	**Paragraph 3 to 8, Table 2, 3, S3 and Figure 1, 2, 3**		
	**38**	Estimates of diagnostic performance and their precision	**Paragraph 3 to 8, Table 2, 3, S3 and Figure 1, 2, 3**		
	**39**	Failure analysis of incorrect results		**No**	
DISCUSSION					
	**40**	Study limitations	**Paragraph 7**		

	**41**	Implications for practice, including intended use and/or clinical role	**Paragraph 1 to 6**		
OTHER INFORMATION					
	**42**	Provide a reference to the full study protocol or to additional technical details		**No**	
	**43**	Statement about the availability of software, trained model, and/or data	**Declaration Section**		
	**44**	Sources of funding and other support; role of funders	**Declaration Section**		

NA = not applicable.

Table S4. Area under the curve, accuracy, balanced accuracy, precision, sensitivity F1-score and kappa score for the radiomic model in the cross-validation in the training set, considering a threshold of 0.5.

Metric	KNN	SVC	MLP	RF	Decision Tree
AUC_mean	0.742	0.790	0.747	0.799	0.654
AUC_std	0.034	0.047	0.036	0.041	0.034
accuracy_mean	0.712	0.740	0.715	0.746	0.667
accuracy_std	0.021	0.032	0.037	0.019	0.030
balanced_accuracy_mean	0.697	0.715	0.698	0.721	0.654
balanced_accuracy_std	0.025	0.037	0.040	0.026	0.034
precision_mean	0.646	0.694	0.644	0.704	0.575
precision_std	0.055	0.057	0.066	0.034	0.066
sensitivity_mean	0.610	0.602	0.617	0.607	0.593
sensitivity_std	0.076	0.062	0.052	0.054	0.044
f1_score_mean	0.622	0.644	0.630	0.652	0.583
f1_score_std	0.037	0.056	0.056	0.044	0.051
kappa_mean	0.742	0.790	0.747	0.799	0.654
kappa_std	0.034	0.047	0.036	0.041	0.034

KNN = K-Nearest Neighbors; MLP = Multi-Layer Perceptron; RF = Random Forest; std = standard deviation; SVC = Support Vector Machine

Table S5. Area under the curve, accuracy, balanced accuracy, precision, sensitivity F1-score and kappa score for the combined radiomic and PI-RADS model in the cross-validation in the training set, considering a threshold of 0.5.

Metric	KNN	SVC	MLP	RF	Decision Tree
AUC_mean	0.803	0.843	0.835	0.853	0.719
AUC_std	0.018	0.038	0.043	0.039	0.033
accuracy_mean	0.756	0.791	0.775	0.796	0.732
accuracy_std	0.029	0.031	0.031	0.028	0.029
balanced_accuracy_mean	0.737	0.778	0.762	0.783	0.719
balanced_accuracy_std	0.029	0.036	0.038	0.032	0.033
precision_mean	0.714	0.740	0.719	0.748	0.660
precision_std	0.026	0.041	0.038	0.044	0.043
sensitivity_mean	0.641	0.719	0.702	0.724	0.660
sensitivity_std	0.051	0.064	0.074	0.053	0.064
f1_score_mean	0.674	0.729	0.708	0.735	0.658
f1_score_std	0.030	0.048	0.049	0.043	0.045
kappa_mean	0.481	0.558	0.525	0.568	0.438
kappa_std	0.055	0.069	0.069	0.062	0.063

KNN = K-Nearest Neighbors; MLP = Multi-Layer Perceptron; RF = Random Forest; std = standard deviation; SVC = Support Vector Machine

Table S6. Area under the curve, accuracy, balanced accuracy, precision, sensitivity F1-score and kappa score for the combined radiomic, PI-RADS and clinical variables model in the cross-validation in the training set, considering a threshold of 0.5.

Metric	KNN	SVC	MLP	RF	Decision Tree
AUC_mean	0.819	0.859	0.848	0.865	0.741
AUC_std	0.023	0.035	0.035	0.036	0.035
accuracy_mean	0.778	0.794	0.791	0.801	0.746
accuracy_std	0.038	0.026	0.022	0.041	0.030
balanced_accuracy_mean	0.757	0.781	0.777	0.788	0.741
balanced_accuracy_std	0.036	0.035	0.031	0.049	0.035
precision_mean	0.747	0.748	0.745	0.752	0.664
precision_std	0.050	0.033	0.024	0.033	0.029
sensitivity_mean	0.663	0.720	0.711	0.733	0.716
sensitivity_std	0.040	0.082	0.075	0.092	0.063
f1_score_mean	0.702	0.731	0.726	0.741	0.689
f1_score_std	0.044	0.047	0.043	0.063	0.042
kappa_mean	0.525	0.564	0.556	0.579	0.474
kappa_std	0.075	0.062	0.052	0.090	0.064

KNN = K-Nearest Neighbors; MLP = Multi-Layer Perceptron; RF = Random Forest; std = standard deviation; SVC = Support Vector Machine

Table S7. Accuracy, balanced accuracy, precision and F1-score for the radiomic, PI-RADS + Radiomics and the combined model for discriminating significant prostate cancer (sPCa) considering a threshold of 0.5.

Classification Model	Accuracy	Balanced Accuracy	Precision	F1-score
Radiomics (RAD)	0.785	0.762	0.748	0.703
PI-RADS - Radiomics (PIR_RAD)	0.811	0.797	0.766	0.749
Radiomics – PI-RADS – Clinical Variables (RAD_PIR_CLIN)	0.828	0.814	0.791	0.770

PI-RADS = Prostate Imaging-Reporting and Data System

Figure S1. Calibration curves of the four trained models alongside the corresponding Brier score and Expected Calibration Error (ECE).

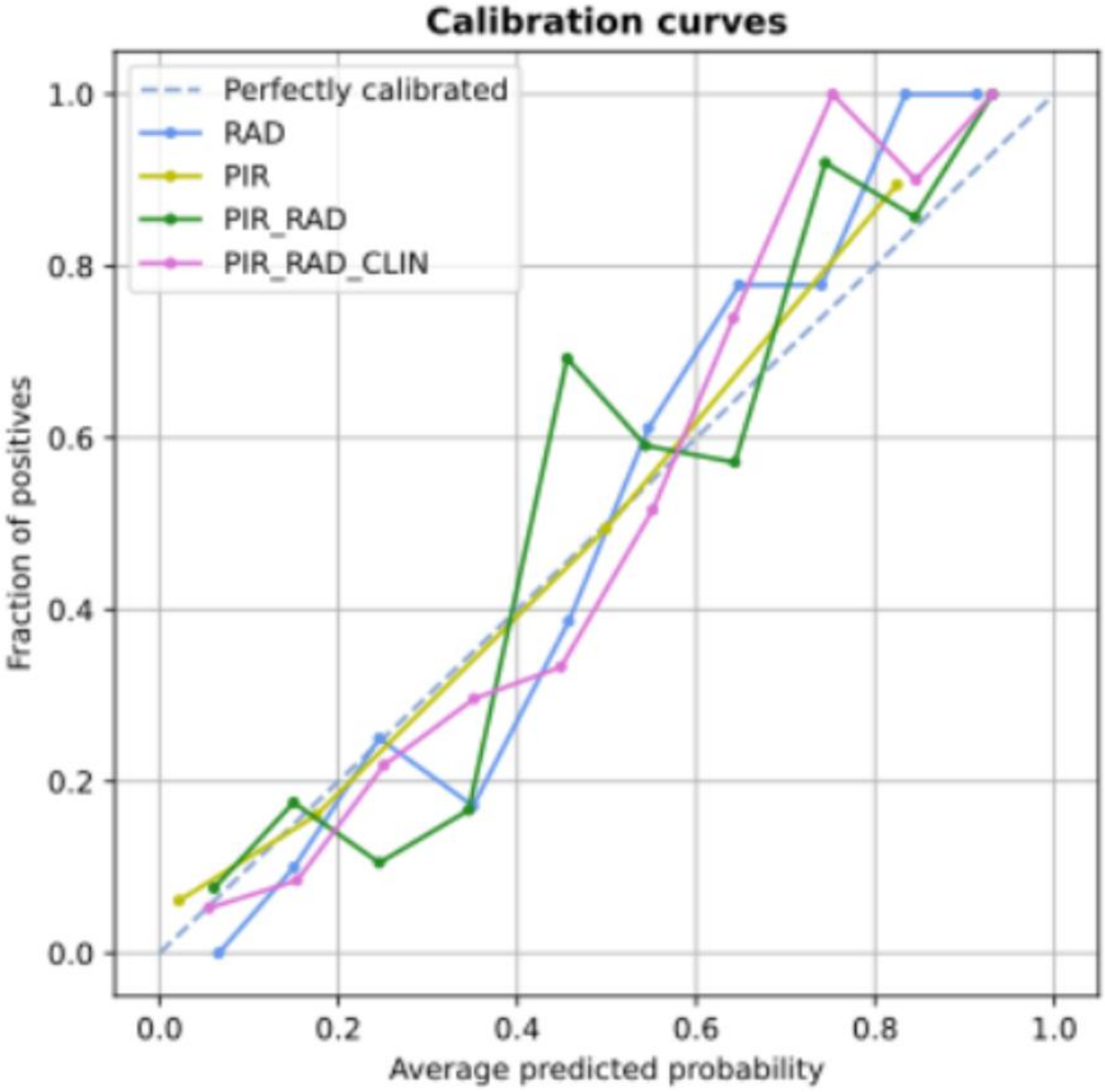

Model	Brier score	ECE
RAD	0.161	0.078
PIR	0.151	0.026
PIR_RAD	0.137	0.075
PIR_RAD_CLIN	0.129	0.067
